# Using Nature-Based Rehabilitation to Restart a Stalled Process of Rehabilitation in Individuals with Stress-Related Mental Illness

**DOI:** 10.3390/ijerph120201928

**Published:** 2015-02-09

**Authors:** Eva Sahlin, Gunnar Ahlborg, Artur Tenenbaum, Patrik Grahn

**Affiliations:** 1 Department of Work Science, Business Economics and Environmental Psychology, Swedish University of Agricultural Sciences, P.O. Box 88, Alnarp S-230 53, Sweden; E-Mail: Patrik.grahn@slu.se; 2 Institute of Stress Medicine, Sweden and Sahlgrenska Academy, University of Gothenburg, Region Västra Götaland, Carl Skottbergs Gata 22B, Göteborg SE-413 19, Sweden; E-Mail: gunnar.ahlborg@vgregion.se; 3 Hälsan & Arbetslivet, Region Västra Götaland, Skaraborgs Sjukhus Skövde, Skövde SE- 541 85, Sweden; E-Mail: artur.tenenbaum@vgregion.se

**Keywords:** Nature-Based Rehabilitation, burnout, depression, anxiety, health care utilization, sick leave

## Abstract

After a period of decrease, sick leave in Sweden due to psychiatric diagnoses is on the increase. The lack of established rehabilitation programmes for patients with stress-related mental disorders (SRMD) has opened up for the use of garden/nature in a multimodal rehabilitation context (Nature-Based Rehabilitation, NBR). Region Västra Götaland (VGR) started an NBR to offer additional rehabilitation for its employees on long-term sick leave due to SRMD, where initial care had not been sufficient. The aim was to explore whether the mental health and well-being of NBR participants had improved at the end of the NBR and at three follow-ups, and to explore the development of sick leave and health care utilization according to the NBR model (*n* = 57) and an occupational health service (OHS) model (*n* = 45). Self-assessment instruments for measuring burnout, depression, anxiety and wellbeing, and data from regional and national registers were used. Results showed decreased scores on burnout, depression and anxiety, and increased well-being scores and significantly reduced health care utilization in the NBR group. A large movement from ordinary sickness benefit to rehabilitation benefit was observed, which was not observed in the OHS group. The two groups were in different rehabilitation phases, which limited comparisons. The results point to beneficial effects of using NBR for this patient group and for enhancing a stalled rehabilitation process.

## 1. Introduction

After a peak in 2002, sick leave spells in Sweden began decreasing. However, during the period 2009 to 2012 this downward trend was interrupted; the number of new cases of illness with psychiatric diagnoses has turned upwards again, and is forecasted to soon reach the same high numbers as in 2005 [1]. Besides the suffering of the individual, this is also of great concern for society as well as employers and companies. The dominating diagnoses in this new wave of sick leaves due to stress-related health problems are adjustment disorder and reaction to severe stress (ICD code F43; about 40%) and depressive episodes (ICD code F32; approximately 30%) [1]. Psychiatric diagnoses are the most prevalent in professions containing mostly women. Employees in health and social care, a large sector where 85% of the employees are women, dominate in this regard. However, in the report from the Swedish Social Insurance Agency [2] from autumn 2013 psychiatric diagnoses forms the largest diagnosis group for both women and men. Sick leaves due to psychiatric disorders are more common among individuals aged 30–49 years [1]. The sickness cases involving psychiatric diagnoses tend to be longer than for other diagnoses, recur more often, and are expected to have implications on long-term absenteeism [1,3]. Mental illness and pain are the most common causes of reduced work ability, and work-related health issues caused by mental strain have become more common in the past 15 years [4].

### 1.1. Stress and Exhaustion Disorder

Long-term exposure to stress can lead to stress-related illness such as depression or fatigue/burnout, anxiety disorder [5], and/or exhaustion disorder (ED, ICD code F43.8) [6,7]. The Swedish diagnosis ED is a medical condition with similarities to burnout. ED is a rather new diagnosis that was formally adopted by the Swedish National Board of Health and Welfare in 2005 [8]. It is characterized by physical and mental exhaustion following a long period of stress exposure, accompanied by symptoms such as somatic problems, decrease in cognitive abilities, depressed mood, and sleep problems, leading to a gradually decreased quality of life as well as a decrease in performance. Depression and anxiety are common co-morbid conditions according to Glise and colleagues [7]. Rehabilitation and return to work (RTW) take a long time [9]. No national guidelines have been elaborated for ED, but guidelines for treatment and rehabilitation for this patient group are proposed in the Rehabilitation Council’s final report [10] and by Region Västra Götaland [11], which both recommend a multimodal approach including physiotherapeutic, psychotherapeutic, and pharmacologic interventions along with prescribed sick leave, combined with occupational therapy and physical exercise. ED patients have traditionally been treated in primary care for some of their symptoms, with limited success [10,12,13].

### 1.2. Nature and Health

A considerable amount of research has explored a wide variety of health effects on humans when exposed to nature environments. These include beneficial effects on cognitive abilities and depleted mental resources [14,15,16], restoration from stress [17,18,19,20], and health [21,22]. Nature experiences have previously been reported to open up for existential reflections, which positively affected the recovery from stress-related mental disorders in an NBR [23].

There are essentially two theories claiming to explain the beneficial effects of interacting with nature: *attention restoration theory (*ART) [24], and *psycho-evolutionary theory* (PET) [25]. ART has a cognitive focus, and claims that depleted mental resources caused by overused and fatigued directed attention can be recovered in a restorative environment comprising the following four essential features: (i) richness in capturing the visitor’s *fascination* in a soft and unchallenging way; (ii) offering a feeling of *being away* from everyday demands; (iii) being *compatible* with the visitor’s current needs; and (iv) having an *extent* that gives a sense of a cohesive whole but also space for discovery [24]. These effects have been explored in several studies [14,15,26,27]. According to PET, contact with nature environments experienced as non-threatening affect an individual both psychologically and physiologically [25]. These effects have also been explored in previous studies [17,28,29]. For a more detailed description of the theories, please see Stigsdotter *et al.* [30].

### 1.3. Nature-Based Rehabilitation

The lack of established rehabilitation programmes for patients with stress-related mental disorders (SRMD) has opened up to a rather new approach: Nature-Based Rehabilitation (NBR), originally developed at the Swedish University of Agricultural Sciences at Alnarp. NBR in Sweden often embraces two parts: (1) traditional medical rehabilitation methods used for SRMD such as relaxation, stress management, physiotherapeutic exercises, body awareness, conversational therapy, and handicraft, all of which are professionally integrated into a nature context; and (2) activities, or simply being, in a garden or/and nature. The number of NBRs addressed to individuals with SRMD has grown rapidly in Sweden in the past ten years. Several studies have reported beneficial health effects related to different types of NBR, for instance the importance of the natural environments in the process of recovery from ED and stress-related mental disorder [23,31,32]. Furthermore, as reported by Pálsdóttir and colleagues [32], a majority of participants (63%) returned to the labour market after having completed an NBR similar to the one in this study. Währborg, Petersson and Grahn [33] reported reduced health care consumption after completed rehabilitation for NBR participants compared to a reference population, but no significant difference for sick leave. Increased self-assessed work ability, as well as reduction in sick leave and reduction in self-assessed burnout, for participants after a nature-based stress management course have previously been reported by Sahlin and colleagues [31]. Nordh, Grahn and Währborg [34] demonstrated beneficial effects on physical and burnout scores during a forest intervention for participants on long-term sick leave due to depression and anxiety disorders. Sonntag-Öström and colleagues [35] reported psychological as well as physiological recovery in individuals with stress-related exhaustion after interventions in the boreal forest in Northern Sweden. Decline in depression severity has been reported for clinically depressed individuals participating in a therapeutic horticultural programme [36,37].

The favourable effects achieved in NBR are explained in the *theory of supportive environments* [38], assuming that recovery from stress-related mental disorders may be enhanced through support from a specially designed garden or a specially selected nature environment, as well as through mild and limited sensory stimulations, and by being in a small group of participants in order to limit social (often demanding) contact. The programme is tailored to meet the needs of the particular targeted group of participants, and is planned and conducted by a multidisciplinary team composed of medical professionals and professionals from the gardening/nature sector.

Because of increasing numbers of employees on long-term sick leave due to stress-related disorders, Region Västra Götaland (VGR), a large public health care organization in Sweden, started an NBR directed at their own employees. The conventional rehabilitation of employees in VGR is handled by the region’s own occupational health service (OHS), where a team-based rehabilitation model has been developed for this patient group. The NBR was started to offer additional rehabilitation efforts for employees on long-term sick leave due to stress-related mental disorders, where initial care had not been sufficient. The individuals’ rehabilitation process had stalled and no progress was observed. It was thus of great interest to explore what had happened to the group participating in this new intervention.

We therefore decided to perform a follow-up study comparing symptoms of burnout, depression, anxiety, and well-being at the start of the NBR with the situation up to twelve months after participation in the programme. We also wished to assess the registered utilization of sickness benefits and health care. Since the NBR was started by VGR as a compliment to the organization’s in-house OHS, we believed it would be interesting to explore the same type of register data among similar patients who had participated in the conventional OHS rehabilitation.

### 1.4. Aim

The main aim of this observational follow-up study was to explore the effects of NBR in patients with exhaustion disorder or stress-related mental disorders.

#### Specific Aims

1. To explore whether the mental health and well-being of NBR participants had improved at the completion of the rehabilitation as well as six and twelve months thereafter, compared with the start of rehabilitation (Aim 1).
2. To explore the development of sick leave and health care utilization after completed rehabilitation according to the NBR model and the OHS model respectively (Aim 2).

## 2. Method

### 2.1. Study Population

**Figure 1:**
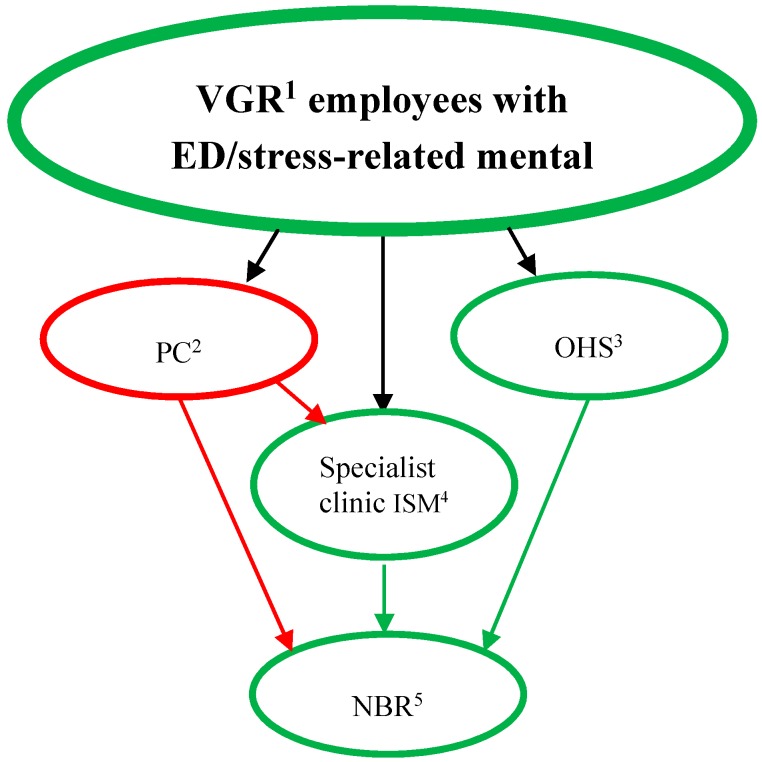
Referral to the NBR of patients with stress-related mental disorders. Green lines indicate activities directly within VGR^1^. Notes: VGR^1^= Region Västra Götaland; PC^2^ = Primary care service; OHS^3^= the VGR Occupational health service; ISM^4^ = Institute of Stress Medicine, VGR.; NBR^5^ = the Nature-Based Rehabilitation started by VGR.

The study participants were all employees at Region Västra Götaland (VGR), and had initially contacted either the in-house OHS or primary care for their mental health problem. Sixteen of the NBR participants were medically examined and treated at the OHS, 13 at a specialist clinic within VGR (Institute of Stress Medicine, ISM), four by private practitioners, and the remaining 24 at primary health care centres (PC) (Figure 1). Participants had achieved rehabilitation according to treatment as usual (PC), to the OHS model, or to a multimodal treatment at ISM.

### 2.2. The NBR Group 

Inclusion criteria for participation in the NBR were, besides employment in VGR, diagnosis regarding stress-related mental illness (such as ED, depression, anxiety) with prolonged sick leave (more than three months), and that participants had reached a level of recovery sufficient to transport themselves to the NBR. Individuals with other serious mental or physical illness were not included. Participants were also not to be suicidal or likely to have substance abuse problems. The participants had been on sick leave for three months to twelve years when they started the NBR (mean 19 months, standard deviation 24). The NBR group was comprised of 57 participants (53 women), who participated in the NBR during October 2007–June 2014. Mean age was 45 years (range 26–63); for women the mean was 44.8 (range 26–63) and for men 52.3 (range 35–62). Prior to acceptance and consenting to participate in the NBR, each participant visited the NBR for an interview with the psychotherapist on the rehabilitation team, and was offered the possibility to ask questions about the NBR on this occasion.

From a total of 72 participants who had completed the NBR during the period, 57 are included in the analysis concerning burnout, depression, anxiety, and well-being. The reasons for excluding the other 15 were: one had died, one could not be reached to be asked about participating in the study, three declined participation in research, one had inappropriate co-morbidity, and nine did not complete the follow-ups, had too many missing data in the questionnaires, or had no baseline data available. The NBR participants had diagnoses covered under the ICD code F43 category (adjustment disorder, F43.2; exhaustion disorder, F43.8; reaction to severe stress unspecified, F43.9), and/or depression (F32), and/or anxiety (F41).

To answer the research questions concerning health care utilization and sick leave, 44 participants (all women) from NBR were included. The discrepancy (57/44) was due to continued collection of data from self-assessment questionnaires six months longer than the time frame for collecting register data for health care utilization and sick leave.

### 2.3. The Occupational Health Service Group (OHS)

As depicted in Figure 1 patients were recruited differently to OHS and NBR and the aim was to explore sick leave and health care utilization separately in the two groups. Even though OHS thus did not serve as a control group we wanted to select these patients to be as similar as possible to the NBR. We thought that this would make the interpretation and discussion of the results more relevant both from an individual and an organizational perspective.

The OHS consisted of 45 female patients (mean age 49, range 32–61) with more than 14 days of prescribed sick leave (before the rehabilitation started) due to ED, reaction to severe stress unspecified, depression or anxiety as described above for the NBR group.

**Figure 2:**
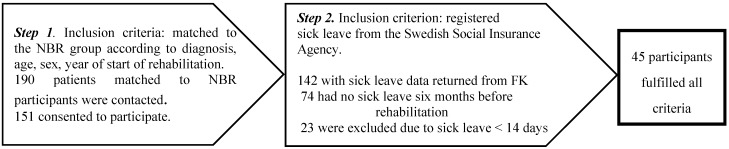
The recruitment process for the OHS group and the inclusion criteria connected to each step.

The selection of the OHS group was done in two steps. First, participants were matched as closely as possible to the NBR group concerning age, sex, diagnosis and year of rehabilitation start, giving 190 possible participants in this group. Of these, 151 consented to participate in the study; however, sick leave data returned from the Swedish Social Insurance Agency included only142 participants (see Figure 2). Sixty-eight of the 142 fulfilled the criteria of sick leave six months before rehabilitation start. However, as the ambition was to make the OHS group more similar to the NBR group with respect to sick leave, we decided to only include participants with more than 14 days of sick leave in the analysis, giving a total number of 45 subjects in the OHS group (see Figure 2).

The study was approved by the regional ethical board in Göteborg, Sweden (Dnr (diary number) 566-12). All participants in the study have signed informed consent after being informed about the study and ethical issues.

### 2.4. Recruitment

#### 2.4.1. The NBR Group

The majority of participants in the NBR group had previously participated in evaluations of the same NBR during October 2007–September 2012. The data from these evaluations were also used for this study, together with data from participants from the NBR from October 2012–June 2014, and additional register data concerning sick leave, health care consumption and diagnosis.

There were two methods of recruitment. Participants who had finished NBR in earlier years were contacted by telephone by the first author, who requested their permission to send them, by post, information about the study, a form for informed consent to participate in the study, and a postage-paid return envelope. These telephone calls gave the participants the opportunity to ask questions about the study.

For the participants still taking part in the NBR, at the end of their rehabilitation the rehabilitation team gave them an information sheet about the study, the form for informed consent, and a sealable envelope for the consent form together with the self-assessment instruments for the first follow-up. Some participants chose to give their signed consent directly to the team, while some sent it to the first author by post in the postage-paid return envelope. After receipt from a participant, the team handed over the completed self-assessment forms to the first author, and (if not mailed) also the envelope including the informed consent form. All data were thereafter stored in a locked research archive. The participants had the opportunity to ask questions about the study during the first author’s visits in the group. The participants were all aware of the first author’s role as an independent researcher not involved in the rehabilitation.

#### 2.4.2. The Group from the Occupational Health Service

The patient register at the OHS was used to identify individuals for possible inclusion in the reference group. This selection procedure was carried out by staff at the OHS. The matched 190 patients were contacted by phone, informed about the study and asked for their consent to use data from their medical records. They were asked for permission to send them by post information about the study, a form for informed consent on participation in the study, and a postage-paid return envelope. For a smaller number of patients (13) not contactable by phone, the information and consent form were sent by post without previous contact.

### 2.5. Two Types of Rehabilitation

#### 2.5.1. Rehabilitation According to the NBR Model

The rehabilitation was led by a multidisciplinary team composed of a gardener and a biologist from the “green” sector, as well as a physiotherapist, an occupational therapist, and a psycho therapist (also trained in art therapy) from the health care sector. The rehabilitation included garden activities based on the current season, weekly guided walks in the nearby nature reserve, therapeutic painting, therapeutic group as well as individual conversations, guided relaxation in nature and indoors (mostly mindfulness and breathing techniques), body awareness, and information about stress and stress reactions and the benefits of physical activity, as well as about nature’s role in health and stress reduction. A majority of the NBR group had psychotherapeutic contact outside the NBR, and most were also receiving pharmacological treatment (mostly SSRI).

This NBR programme was divided into two parts: 16 weeks of rehabilitation, followed by twelve weeks of gradual return to work or study with more and more time at work and a corresponding decrease in participation in the NBR (Table 1).

**Table 1:** Description of the Nature-Based Rehabilitation with respect to rehabilitation time, group size, inclusion and exclusion criteria, professionals on the team, types of environment, and activities within the programme.

The Nature-Based Rehabilitation	Description
Length of rehabilitation	Phase 1: 16 weeks: four days/week, three hours/day
	Phase 2: 12 weeks: gradual return to work or study with corresponding decrease in participation in NBR
Group size	Maximum eight individuals
Inclusion criteria	Employed by the Region Västra Götaland Stress-related mental disorder Capable of transporting oneself to the NBR
Exclusion criteria	Alcohol addiction Fibromyalgia More severe psychiatric diagnosis Suicidal risk Physical handicap preventing full participation
Professionals on the team “Green” “Therapeutic”	Biologist/nature guide
	Gardener
	Occupational therapist
	Psychotherapist
	Physiotherapist
Type of “green” environment	Garden and greenhouse
	Wild, tended nature and park environment
“Green” activities	Garden activities
	Guided nature walks
	Handicraft with material from nature
Other activities/content	Therapeutic painting
	Supportive conversations in group or individually
	Relaxation/body awareness
	Information about stress, health and lifestyle

##### The Venue

The site for the NBR bordered an allotment area, a small brook, and a 222-acre nature reserve. The venue consisted of a small house with a conservatory, a garden, and a greenhouse.

#### 2.5.2. Rehabilitation According to the OHS Model

The rehabilitation at the OHS followed a team-based method entailing separate assessments of the patient made by a nurse, physician, physiotherapist and psychologist. This rehabilitation model did not include any garden or nature content. After interviews and examinations of the patient, the different professionals on the team made an overall assessment of the patient based on the information obtained from the team members. Individuals in the reference group received an individually planned rehabilitation based on five key principles: increased physical activity (prescribed), counselling, medication, individual-adapted prescribed complete or partial sick leave, and close dialogue with the employer/manager to define the tasks and adaptation of work to facilitate return to work as early as possible, without risking a worsening or recurrence of disease. Forty-five participants in the reference group fulfilled the sick leave criteria of at least one month of sick leave during the six months preceding the rehabilitation.

### 2.6. Measures

When the NBR model was designed, the VGR management decided to evaluate the health effects of this intervention. Instruments for evaluating participants’ mental health and well-being were chosen according to recommendations by the ISM, a research and specialist clinic within VGR for these types of stress-related disorders. These instruments, described in the following section, were not used at the OHS, however.

#### 2.6.1. Burnout

Nature’s role has been documented in previous studies when it comes to restoring mental and emotional exhaustion [24,35,39] and cognitive weariness [27] hence, the Shirom-Melamed Burnout Questionnaire (SMBQ) was chosen to measure burnout. This self-assessment questionnaire consists of four subscales, two of which are included in Shirom’s [40,41] definition of burnout: emotional and physical exhaustion (eight items) and cognitive weariness (six items). The other two subscales are tension (four items) and listlessness (four items). The SMBQ consists of 22 items, all with a seven-point response scale (1 = almost never, 7 = almost always). The Swedish version of the SMBQ correlates highly with the Maslach Burnout Inventory [12], and has been used to evaluate treatment effects [42].

We calculated the mean for the total score and a cut-off at 4.4 to create a dichotomous variable indicating burnout or not, based on recommendations in a newly conducted validation study by Lundgren-Nilsson and colleagues [43], which also confirmed that the instrument can be used as an overall measure of burnout/stress-related fatigue.

#### 2.6.2. Depression

The Beck Depression Inventory (BDI-II) [44] is an established questionnaire for measuring degree of and changes in depression. The instrument consists of 21 groups of symptoms and attitudes, which are assessed by the individual on a four-point scale valued 0–3 in terms of severity (0 = the most positive response alternative and 3 = the most negative response alternative) with a maximum possible 63 points. The scores are interpreted in relation to the chosen cut-off values: 0–13 points (minimal depression); 14–19 points (mild depression); 20–28 points (moderate depression); 29–63 points (severe depression). The BDI has been used in a large number of intervention studies, including physical exercise as treatment for depression [45].

#### 2.6.3. Anxiety

The Beck Anxiety Inventory [46] is an established questionnaire for the self-assessment of degree of anxiety and changes in anxiety states. It includes 21 claims/symptoms, which are estimated by the individual on a four-point scale valued 0–3 in terms of severity (0 = not at all, 3 = severely-it bothered me a lot) with a maximum possible 63 points. The scores are interpreted in relation to the chosen cut-off values: 0–7 points (minimal level of anxiety); 8–15 points (mild anxiety); 16–25 points (moderate anxiety); 26–63 points (severe anxiety).

#### 2.6.4. Well-Being

The Psychological General Well-Being Index (PGWB) [47] is a quality of life instrument constructed to measure self-assessed well-being. It consists of 22 questions/statements divided into six subscales: health, self-control, mood, anxiety, vitality and positive well-being. Each item has six response options, scored from 1 (the most negative value) to 6 (the most positive value), with a total score range between 22 and 132; the higher score the better the well-being. The PGWB has good reliability and validity compared to other established methods for measuring mental well-being.

The instrument has previously been used to explore quality of life in patients with various symptoms and between patients receiving different therapies, and to evaluate a variety of treatments. It has proven to be sensitive to clinically relevant changes [48]. The PGWB has thus been used to compare groups or to measure the effects of an intervention/treatment on the subjective experience of well-being. A recently published validation study of the PGWB concludes that the instrument is suitable for monitoring well-being during intervention for ED/burnout [49].

**Figure 3:**
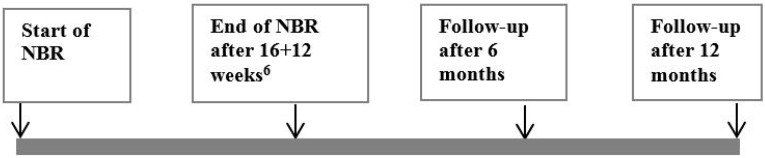
Time frame for the collection of data by self-assessment instruments from the participants in the Nature Based Rehabilitation. Note^6^: 16 weeks of rehabilitation followed by 12 weeks of gradual return to work or study.

SMBQ BDI-II, BAI, and PGWB were distributed only to the NBR participants and at four occasions: at the start of NBR (baseline measure), and at three follow-ups at the end of the two periods (16 weeks of rehabilitation followed by12 weeks of gradual return to work or study) included in the NBR, and six and twelve months thereafter (Figure 3).

#### 2.6.5. Sick Leave

Register data from the Swedish Social Insurance Agency’s statistics were collected to compare the sick leave and compensation for participating in occupational rehabilitation (henceforth referred to as rehabilitation compensation) for both study populations (NBR and OHS) for the periods six months before starting rehabilitation, and six and twelve months after finishing the 16 weeks of rehabilitation. The number of days with full- or part-time sick leave/rehabilitation compensation were collected and calculated. *Rehabilitation compensation* is a type of benefit granted to an individual when he/she has been judged to be sufficiently rehabilitated so that he/she can participate in work training, in a work-oriented rehabilitation programme or studies. The rehabilitation must be a part of a rehabilitation plan developed in cooperation with the Social insurance agency.

#### 2.6.6. Health Care Utilization

From VGR’s register of health care utilization (VEGA), data for number of visits, type of health care contact, and reason for the visit were collected. However, as the OHS did not report to VEGA, responsible staff at the OHS retrieved equivalent and complementary data from the patient records. The number of visits to the doctor, psychologist, physiotherapist and nurse were merged into one variable covering visits to all these medical professionals. The three periods for measuring sick leave and health care utilization are:

Period 1 (P1): six months before rehabilitation to starting the 16 weeks of rehabilitation.

Period 2 (P2): from completion of the 16 weeks of rehabilitation and six months ahead.

**Figure 4:**
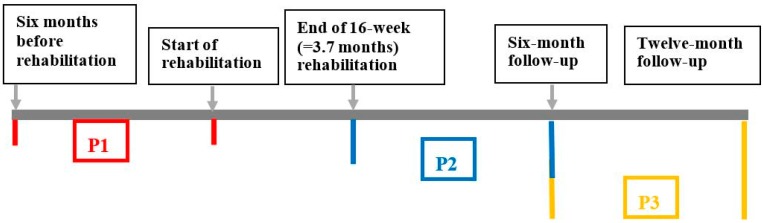
The time frames for the measuring periods concerning health care utilization and sick leave six months before rehabilitation start, and six and twelve months after completion of rehabilitation.

Period 3 (P3): from completion of the 16 weeks of rehabilitation and seven to twelve months thereafter (Figure 4).

### 2.7. Statistical Analysis

Descriptive statistics are presented as means and standard deviations (continuous variables). Differences in proportion with 95% confidence intervals (CI) between the baseline measures at start of NBR and each of the follow-ups (end of NBR and at six and twelve months) were calculated for SMBQ < 4.4 according to the method suggested by Newcombe [50]. Although SMBQ items have response scales of ordinal character, mean values with standard deviation are often reported in the literature. Therefore, the SMBQ results in this study will be presented in this customary manner as well as in the way described above.

The PGWB raw scores were transformed into metric values according to Lundgren-Nilsson and colleagues [49], thus making it possible to use the transformed scores as an interval scale. The *t*-test for paired samples was used to analyse the differences in PGWB scores between the baseline measures at start of NBR and each of the follow-ups (end of NBR and at six and twelve months). The Wilcoxon Signed Rank test was used to analyse BDI-II and BAI. All analyses were performed using IBM SPSS (Statistical Package for the Social Sciences**)** Statistics version 22 (SPSS Inc., Chicago, IL, USA)

## 3. Results

### 3.1. Mental Health

#### 3.1.1. Burnout

Mean burnout scores decreased from 5.2 (SD 0.88) at start of NBR rehabilitation to 4.4 (SD 1.16) at the end of NBR, 4.26 (SD 1.28) at six months, and 4.12 (SD 1.26) at twelve-month follow-up. The differences in the proportion scoring below the cut-off of 4.4 between start and all three follow-ups were statistically significant (Table 2).

**Table 2:** Differences in paired proportions, with 95% confidence interval (CI) for participants scoring ˂4.4 on the SMBQ^7^. Comparing start of course with follow-up at course end, and six and twelve months, respectively.

Length of Follow-Up	Start	Follow-Up	Difference	95% CI
	*%*	*%*		
SMBQ ^7^				
End of course (*n* = 51)	23.5	47.1	23.5	8.6; 36.9
6 months (*n* = 46)	23.9	47.8	23.9	7.9; 38.2
12 months (*n* = 40)	22.5	62.5	40.0	19.6; 55.8

Note:^7^ The Shirom-Melamed Burnout Questionnaire assesses emotional and physical. exhaustion, cognitive weariness, listlessness and tension.

#### 3.1.2. Depression

**Figure 5:**
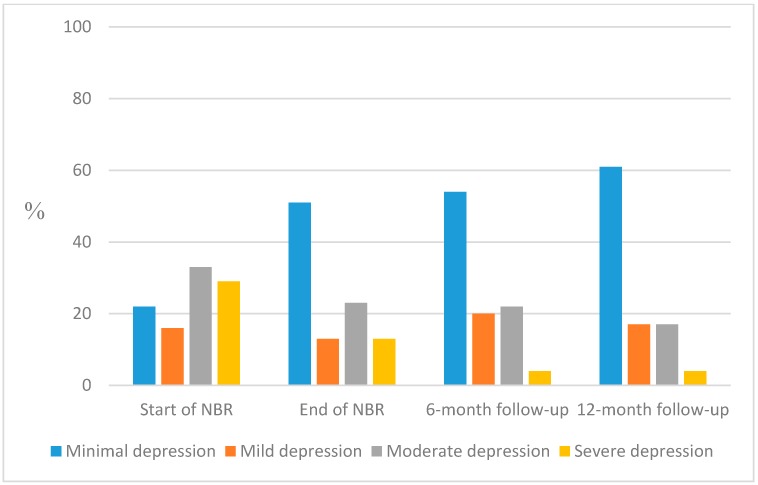
Proportions of participants with grand sum according to the standardized cut-offs for BDI-II: minimal (0–13), mild (14–19), moderate (20–28) and severe depression (29–63) measured at start of NBR and follow-up at end of NBR, and after six and twelve months, respectively.

Mean value of BDI-II at start of NBR was 23.2 (SD = 10.0), and at the three follow-ups 15.6 (SD = 8.7), 14.2 (SD = 8.0) and 13.0 (SD = 8.7), respectively, showing a movement from moderate to mild depression for the group. The number of participants scoring “moderate” or “severe” depression decreased from 52% (divided into: moderate 29% and severe 33%) at start of NBR to 26% (divided into: moderate 22% and severe 4%) at six-month follow-up, and had decreased further to 21% at twelve-month follow-up (divided into: moderate 17% and severe 4%) (Figure 5). 

Most participants (88%) lowered their depression score after rehabilitation. Analyses were performed with Wilcoxon Signed Rank test and all comparisons showed statically significant decreases at each follow-up compared to start of NBR (*p* < 0.0001; Table 3).

**Table 3:** Comparisons of the rank scores for BDI-II; comparing start of NBR with follow-up at the end of NBR and at six- and twelve-month follow-up, respectively.

Length of Follow-Up	Start
	*Z(p)*
End of NBR (*n* = 52)	−4.9 (*p* < 0.0001)
6 months (*n* = 48)	−4.7 (*p* < 0.0001)
12 months (*n* = 43)	−4.5 (*p* < 0.0001)

#### 3.1.3. Anxiety

Mean value on the BAI at start of NBR was 17.2 (SD 11.8), and for the three follow-ups 12.8 (SD 10.1), 12.1 (SD 8.4) and 10.2 (SD 7.8), respectively, showing a movement from moderate to mild anxiety for the group.

**Figure 6:**
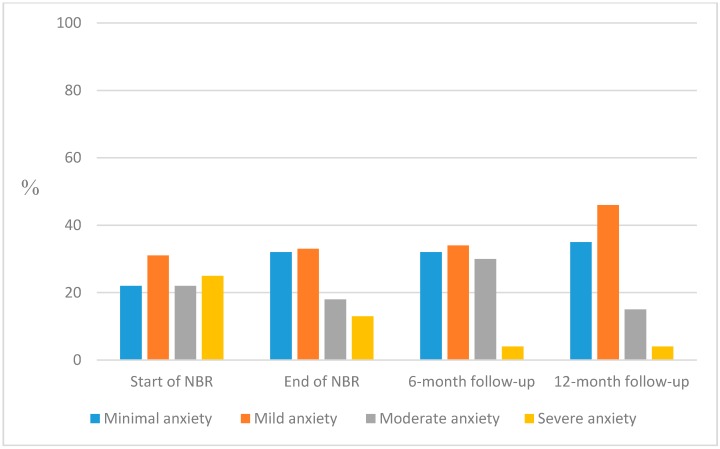
Proportions of participants with grand sum according to the standardized cut-offs for BAI: minimal (0–7), mild (8–15), moderate (16–25) and severe (26–63) anxiety measured at start of NBR and follow-up at end of NBR, and after six and twelve months, respectively.

The number of participants scoring “moderate” or “severe” anxiety decreased from 47% (divided into: moderate 22% and severe 25%) at start of NBR to 34% (divided into: moderate 30% and severe 4%) at six-month follow-up, and had decreased further to 19% (divided into: moderate 15% and severe 4%) at twelve-month follow-up (Figure 6).

Most participants (63%–71%) lowered their anxiety score after rehabilitation. Irrespective of whether analyses were performed with Wilcoxon Signed Rank test and all comparisons showed statically significant decreases at each follow-up compared to start of NBR (*p* < 0.0001–0.005; Table 4).

**Table 4:** Comparisons of the rank scores for BAI; comparing start of NBR with follow-up at the end of NBR and at six- and twelve-month follow-up, respectively.

Length of Follow-Up	Start
	*Z(p)*
End of NBR (*n* = 49)	−3.4 (*p* =0.001)
6 months (*n* = 48)	−2.8 (*p* =0.005)
12 months (*n* = 43)	−3.7 (*p* <0.0001)

#### 3.1.4. Well-Being

**Figure 7:**
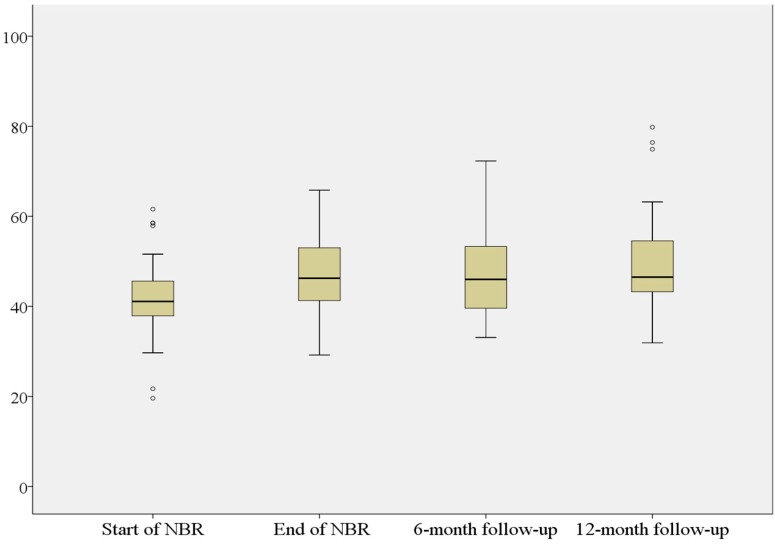
PGWB**^8^** metric scores at start of NBR, at the end of NBR and at six- and twelve-month follow-up. Note: PGWB ^8^ the Psychological general well-being assesses health, self-control, mood, anxiety, vitality and positive well-being.

Mean value on the PGWB at start of NBR was 41.9 (SD = 8.1), and for the three follow-ups 46.7 (SD = 8.8), 47.8 (SD = 9.4) and 49.1 (SD = 10.7), respectively, showing a gradual increase in mean values for the PGWB scores, indicating improvement in the participants’ self-assessed well-being (Figure 7).

All comparisons showed statistically significant improvement (*p* < 0.0001; Table 5).

**Table 5:** Comparison of mean differences for PGWB score; comparing start of NBR with follow-up at the end of NBR and at six- and twelve-month follow-up, respectively.

Length of Follow-Up	Start	Follow-Up	t(df)	*p*-value
	*Mean (SD)*	*Mean (SD)*		
PGWB^4^ Score				
End of NBR (*n* = 52)	41.9 (8.2)	47.0 (9.0)	−4,85 (51)	<0.0001
6 months (*n* = 48)	41.9 (8.4)	47.9 (9.4)	−4.27 (47)	<0.0001
12 months (*n* = 43)	41.7 (8.4)	49.5 (11.0)	−4.45 (42)	<0.0001

### 3.2. Sick Leave and Rehabilitation Compensation

All participants (NBR and OHS) received sick-leave compensation before starting rehabilitation, and more than 90% also did so to some extent during the follow-up periods (P2 and P3). For the NBR group, a decrease in total number of days with sick leave compensation (partial or full) was observed (Table 6). The differences in mean values between P1 and P2 were statistically significant (mean difference 42.5, CI 24.5; 60.5, *t* = 4.8; df = 43; *p* < 0.0001), as were those for P2 and P3 (mean difference 30.8, CI 6.0; 55.5, *t* = 2.5; df = 43; *p* = 0.016). On the other hand, there was an increase in number of days with rehabilitation compensation, which 71% of this group were receiving during the last follow-up period, and the differences in mean values between P1 and P2 were statistically significant (mean difference 44.7, CI −58.9; −30.5, *t* = −6.4; df = 43 *p* < 0.0001). Although a decrease in number of days with rehabilitation compensation was observed between P2 and P3, this was not enough to reach statistically significant difference (Table 6).

**Table 6:** The total number of days with sickness and rehabilitation compensation (partial or full) for participants in the NBR group (*n* = 44) during P1^1^, P2^2^, and P3^3^.

Total Number of Days of Partial or Complete Sick Leave Compensation and Rehabilitation Compensation	P^1^ = 6 Months Before Rehabilitation Start	P^2^ = 6 months after Completed Rehabilitation	P^3^ = 7–12 Months after Completed Rehabilitation
*-sick leave*	7204	5335	3982
*-rehabilitation compensation*	16	1983	1379

Contrary to the NBR group, the OHS group showed an increase in total number of days with sick leave compensation (Table 7), and the difference was statistically significant between P1 and P2 (mean difference 68.9; CI −93.0; −44.8, *t* = −5.8; df = 44; *p* < 0.0001). A decrease in number of sick leave days was observed for P3, and the difference was statistically significant between P2 and P3 (mean difference 67.7; CI 44.8; 90.6; *t* = 5.6; df = 44; *p* < 0.0001). As expected, there were no days with rehabilitation compensation before start of rehabilitation in this group. It was not until P3 that this type of compensation became more frequent (Table 7), but even then it was still being received by only 13% of the OHS participants. The difference between P2 and P3 did not reach statistical significance (*p* = 0.063).

**Table 7:** The total number of days with sickness and rehabilitation compensation (partial or full) for participants in the OHS group (*n* = 45) during P1^1^, P2^2^, and P3^3^.

Total Number of Days of Partial or Complete Sick Leave Compensation and Rehabilitation Compensation	P^1^ = 6 Months before Rehabilitation Start	P^2^ = 6 Months after Completed Rehabilitation	P^3^ = 7–12 Months after Completed Rehabilitation
*-sick leave*	3897	6997	3951
*-rehabilitation compensation*	0	50	481

As expected, there were no days with rehabilitation compensation before start of rehabilitation in this group. It was not until P3 that this type of compensation became more frequent (Table 6), but even then it was still being received by only 13% of the OHS participants. The difference between P2 and P3 did not reach statistical significance (*p* = 0.063).

### 3.3. Health Care Utilization

For the NBR group the mean number of visits for health care consumption decreased, comparing six months before rehabilitation (P1) to the follow-up for the six-month period after completed rehabilitation (P2). This difference was statistically significant (mean difference 8.4; CI −12.0; −4.7, *t* = 4.6; df = 43; *p* < 0.0001). The visits remained at the same level for the last follow-up period (P3) (Table 8). For the OHS group the mean number of visits increased when P1 and P2 are compared, but the difference did not reach statistical significance (mean difference 4.3; CI −8.9; 0.26; *t* = −1.9; df = 44; *p* = 0.064). However, the mean number of visits decreased between P2 and P3, showing a statistically significant difference (mean difference 6.6; CI 3.7; 9.4, *t* = 4.6, df = 44, *p* < 0.0001). See Table 9 for mean values and SD for health care utilization.

**Table 8:** Visits to medical professionals for the NBR group displaying mean values and standard deviation (SD) for the measures during P1^1^, P2^2^, and P3^3^.

Visits to Medical Professionals *Mean (SD)*	P^1^ = 6 Months before Rehabilitation Start	P^2^ = 6 Months after Completed Rehabilitation	P^3^ = 7–12 Months after Completed Rehabilitation
NBR *n* = 44	19.2 (11.4)	10.8 (8.9)	10.7 (9.4)

**Table 9:** Visits to medical professionals for the OHS group, displaying mean values and standard deviation (SD) for the measures during P1^1^, P2^2^, and P3^3^.

Visits to Medical Professionals *Mean (SD)*	P^1^ = 6 Months before Rehabilitation Start	P^2^ = 6 Months after Completed Rehabilitation	P^3^ = 7–12 Months after Completed Rehabilitation
OHS *n* = 45	13.2 (15.0)	17.6 (11.5)	11.0 (9.6)

## 4. Discussion and Conclusions

The main findings in this study were that participants in NBR showed decreased scores of self-assessed burnout, depression, anxiety, and increased scores of well-being at all follow-ups compared to start of rehabilitation. A significant reduction in health care utilization after rehabilitation was also observed. Additionally, a large proportion of the participants increased their level of activity by moving from ordinary sickness benefit to rehabilitation benefit, an important step towards return to work. Considering the fact that the NBR group comprised employees with severe stress-related mental disorders, leading to a history of long-term sick leave despite initial treatment by ordinary health care providers, these results seem very promising, for the individual as well as from an organizational and societal perspective. The exploration of register data on sickness benefits and utilization of care among patients entering the rehabilitation provided by the in-house OHS as well indicated that some were still in need of additional efforts twelve months after finishing the usual rehabilitation programme, in order to be able to return to work. Thus, it seems that the investment of the organization in NBR as a compliment to the conventional OHS may also be rewarding from an employer’s perspective.

What made this restart of the rehabilitation process so seemingly successful? The nature and garden content of the NBR comprised a large part of the time (42%) in the weekly schedule, and we suggest that this played a significant part in the participants’ improvement in mental health. The regular exposure to nature for a rather long time (approximately 28 weeks) may have been decisive, for example, in more thoroughly restoring depleted cognitive resources, reducing stress, and incorporating the benefits of the outdoor relaxation, physical activities and daylight exposure, and the many opportunities for reflection due to experiences of soft fascination. This long exposure may have resulted in positive effects of more profound and long-lasting character. Some studies have demonstrated the beneficial effects on mental health of longer nature exposure, which may support this interpretation [23,31,32,36,37,39]. However, Nordh *et al.* [34] showed improvement in burnout scores but not in well-being, depression or anxiety at follow-up after a ten-week forest intervention.

Enhanced emotional balance and well-being through nature exposure have been reported [51,52], offering further support for the interpretation of the results of our study as having a connection to nature experiences.

The education about nature during the guided walks may also have played an important part in the observed improvement in mental health among the participants. Possibilities to distance oneself from a problematic health situation and focus on some interesting detail in nature allowed space for new perspectives concerning one’s recovery as well. The expectation of a positive effect made individuals more prone to go out into nature again. This is in line with findings by Johnsen and Rydstedt [51], showing a stronger inclination to use nature for well-being after having previously experienced positive nature experiences and their effects. It is also in line with Kaplan’s reported [53] claim that education about health benefits from contact with nature may enhance the restorative effect in a restorative environment.

The NBR also offered a context of social coherence, which would be beneficial to people who have spent a long time in the home on sick leave. Being treated with tolerance and acceptance by a professional rehabilitation team and other participants in the same situation as oneself has earlier been shown to be of importance in the recovery of individuals with ED and other stress-related mental problems [23,31].

For the NBR group the number of days with partial or full sick leave continuously decreased from baseline to P2 and P3, and a corresponding increase in days with rehabilitation compensation was observed for the NBR group, indicating that the majority (71%) were involved in occupational training, had started to return to work (RTW) or were studying. For the OHS group the results were quite different, with a substantial increase in days with sick leave benefits for P2 and a decrease during P3, but with no substantial increase in rehabilitation compensation. This indicated that there were several participants in the OHS group who had not reached a recovery level to start RTW at P3.This may partially be explained by the fact that the OHS group were in an earlier phase of their stress-related disorder, some of them being less severely ill and thus not in need of rehabilitation compensation, while others needed a longer time to recover and could eventually become candidates for NBR.

The benefits of a strong and continuous coaching for the NBR participants available four days/week may have facilitated return to activity. The close involvement with manager/workplace during the whole rehabilitation may also have contributed to this, but such contacts were also prevalent during the OHS rehabilitation. This is in line with Sandahl and colleagues’ [54] suggestions for promoting RTW for this patient group, including engagement by the manager/supervisor, gradual return, and group treatment. Strindlund and Ekberg [55] also showed the importance of support from employers/managers, which was reported as significant by 80% of individuals for a successful RTW after long-term sick leave. However, weekly coaching to support the next step after completing a ten-week forest intervention did not lead to work or any further work-related related activities according to Nordh and colleagues [34]. Perhaps again, the long time with firm support in the NBR in this study may be one explanation for the successful results in this respect.

A similar pattern was noted for health care utilization as for sick leave. Interestingly, the visits to medical professionals decreased significantly for P2 and P3 compared to P1.

Comparing the results for sick leave and health care utilization, in both cases P1 has the highest values for the NBR group and P2 for the OHS group, indicating that the two groups are in different phases when starting the respective rehabilitation process**.** The participants in the OHS as well as in the NBR group have the same employer, are highly educated and described as high performing professionals. According to the OHS professionals' experiences, the participants involved in the OHS group are equally motivated to participate in the rehabilitation process to regain health and work as those in the NBR group.

### 4.1. Strengths and Limitations

A strength of this study is that the results are based on validated instruments and data from regional and national registers; furthermore, twelve months of follow-up is a longer time frame than several other studies have used to follow up effects. There were established clinically relevant cut-off levels for all instruments for measuring mental health except for the PGWB. However, research using the PGWB has shown that a difference of four to seven points can be used as a measure of clinically relevant changes in well-being [48].These figures, however, are not based on the metric scores but rather the raw PGWB scores, which in this case showed improvements of twelve points or more. All comparisons between baseline and follow-ups showed clinically relevant changes; thus, well-being improved for the NBR group. However, as these values do not reach the normal values (100–105 points) reported in previous studies [56,57], this is a group in which some individuals are still not experiencing very good well-being.

It may be regarded as a limitation that the study has an explorative design. In order to make more firm conclusions about the effectiveness of NBR compared to conventional rehabilitation without nature content, a randomized, controlled design would have been preferable. Our initial ambition was to have a reference group similar to the NBR group, but it became obvious that the only possibility available within the organization at the time was to observe patients going through the rehabilitation provided by the OHS. A comparison could therefore only be performed with the differences between the groups in mind, as discussed above. The study samples were rather small, which resulted in somewhat uncertain estimates, as indicated by the wide confidence intervals. Many of the changes in the health parameters were clearly statistically significant, however. It is desirable that future studies use a controlled design, comparing an NBR intervention with a comparable rehabilitation programme without nature/garden content, and with a larger study population.

### 4.2. Conclusions

All the results of this explorative study show that the additional rehabilitation intervention including nature/garden (NBR) may reinforce the rehabilitation of individuals with stress-related mental illness who are still on long-term sick leave after having received initial rehabilitation but who have not made progress. Besides the positive effects it has for the individual to move along in the RTW process, this is of great economic value from organizational and societal perspectives as well. We find it likely that a larger part of the participants in NBR with very long-term sickness absence would not have started their RTW without an effective intervention. Also, the proportion of similar patients who had received the standard rehabilitation by the organization’s in-house OHS but were still on sick leave a year later could be candidates for NBR. Firm multidisciplinary support on a regular basis, the group design of the NBR and the environmental support from nature and garden, collectively, seem to be a valuable aid towards improved mental health and RTW, as well as in reducing health care utilization.
