# Correction: Sahlin, E., *et al.* Using Nature-Based Rehabilitation to Restart a Stalled Process of Rehabilitation in Individuals with Stress-Related Mental Illness. *Int. J. Environ. Res. Public Health* 2015, *12*, 1928–1951

**DOI:** 10.3390/ijerph120606946

**Published:** 2015-06-17

**Authors:** Eva Sahlin, Gunnar Ahlborg, Artur Tenenbaum, Patrik Grahn

**Affiliations:** 1 Department of Work Science, Business Economics and Environmental Psychology, Swedish University of Agricultural Sciences, P.O. Box 88, Alnarp S-230 53, Sweden; E-Mail: patrik.grahn@slu.se; 2 Institute of Stress Medicine, Sweden and Sahlgrenska Academy, University of Gothenburg, Region Västra Götaland, Carl Skottbergs Gata 22B, Göteborg SE-413 19, Sweden; E-Mail: gunnar.ahlborg@vgregion.se; 3 Hälsan & Arbetslivet, Region Västra Götaland, Skaraborgs Sjukhus Skövde, Skövde SE- 541 85, Sweden; E-Mail: artur.tenenbaum@vgregion.se

The authors wish to make the following corrections to their paper published in the *International Journal of Environmental Research and Public Health* [1]:

Page 1939, 4 lines from the bottom of the page, the sentence: “The number of participants scoring “moderate” or “severe” depression decreased from 52% (divided into: moderate 29% and severe 33%) at start of NBR to 26% (divided into: moderate 22% and severe 4%) at six-month follow-up, and had decreased further to 21% at twelve-month follow-up (divided into: moderate 17% and severe 4%) (Figure 5)” should read: “The number of participants scoring “moderate” or “severe” depression decreased from 62% (divided into: moderate 33% and severe 29%) at start of NBR to 26% (divided into: moderate 22% and severe 4%) at six-month follow-up, and had decreased further to 21% at twelve-month follow-up (divided into: moderate 17% and severe 4%) (Figure 5)”.

The authors would like to apologize for any inconvenience caused to readers by these changes.
